# Recombinant proteins as promising antigens applied to the immunodiagnosis of Chagas disease: a scoping review

**DOI:** 10.3389/fmicb.2024.1420226

**Published:** 2024-07-30

**Authors:** Carlos Ananias Aparecido Resende, Anna Júlia Ribeiro, Isadora Braga Gandra, Kamila Alves Silva, Lucas da Silva Lopes, Isabelle Caroline dos Santos Barcelos, Carolina Alves Petit Couto, Mariana Teixeira de Faria, Sabrina Paula Pereira, Sandra Rodrigues Xavier, Juliana Martins Machado, Mariana Campos da Paz, Ana Thereza Chaves, Eduardo Antônio Ferraz Coelho, Rodolfo Cordeiro Giunchetti, Miguel Angel Chávez-Fumagalli, Walderez Ornelas Dutra, Ana Alice Maia Gonçalves, Alexsandro Sobreira Galdino

**Affiliations:** ^1^Microorganism Biotechnology Laboratory, National Institute of Science and Technology on Industrial Biotechnology (INCT-BI), Federal University of São João Del-Rei, Divinópolis, Brazil; ^2^Laboratory of Bioactives and Nanobiotechnology, Federal University of São João Del-Rei, Divinópolis, Brazil; ^3^Postgraduate Program in Health Sciences, Infectious Diseases and Tropical Medicine, Faculty of Medicine, Federal University of Minas Gerais, Belo Horizonte, Brazil; ^4^Laboratory of Biology of Cell Interactions, Department of Morphology, National Institute of Science and Technology on Tropical Diseases (INCT-T), Federal University of Minas Gerais, Belo Horizonte, Brazil; ^5^Computational Biology and Chemistry Research Group, Vicerrectorado de Investigación, Universidad Católica de Santa María, Arequipa, Peru

**Keywords:** Chagas disease, *Trypanosoma cruzi*, diagnosis, recombinant, serodiagnosis, immunodiagnosis

## Abstract

Chagas disease (CD), caused by the protozoan *Trypanosoma cruzi*, is an important public health problem, occurring mainly in Latin America. The disease has a major social and economical effect, negatively impacting the life of the infected individuals, and bringing great costs to public health. An early and accurate diagnosis is essential for administration of early treatment. In addition, prognostic tests may aid disease management, decreasing hospitalization costs. However, the serological diagnostic scenario for CD still faces several challenges, making the development of new diagnostic kits a pressing matter. Facing this scenario, several researchers have expanded efforts in developing and testing new antigens, such as recombinant proteins and recombinant multiepitope proteins, with promising results. These recombinant antigens offer several advantages, such as improved sensitivity and specificity, in addition to facilitated scaling. Also, it has been possible to observe a rising number of studies using ELISA and point-of-care platforms, employing these antigens in the past few years. Among them, recombinant proteins were the most applied antigens, demonstrating great capacity to discriminate between positive and negative samples. Although fewer in number, recombinant multiepitope proteins also demonstrated an improved diagnostic performance. Indeed, a great number of studies employing these antigens showed sensitivity and specificity values above 90%, greatly impacting diagnostic accuracy. Nevertheless, despite the good results found, it is still possible to observe some bottlenecks in the development of new antigens, such as the scarcity of tests with sera from the acute phase and the variability of results in different geographic areas. In this sense, aiming to contribute to control and health programs, the continuous search for a more accurate serological diagnosis is essential, both for the acute and chronic phases of the disease.

## 1 Introduction

Chagas disease (CD), caused by the hemoflagellate protozoan *Trypanosoma cruzi* (Chagas, 1909), is a neglected tropical disease considered one of the foremost parasitic infections in the world (Suescún-Carrero et al., 2022). Parasite transmission occurs through several routes, including vector, blood transfusion, organ transplants, food consumption, and transmission from mother to child during gestation (Echeverria and Morillo, 2019; Norman and López-Vélez, 2019; Montoya et al., 2022). The disease is endemic to the Americas, with a wide geographic distribution from central Argentina to the southern United States (Balouz et al., 2017). Currently, it is estimated that 6 million people are infected worldwide (PAHO, 2024). Among Latin American countries, Bolivia, Paraguay, Argentina, and Brazil have a high number of cases, with most *T. cruzi*-infected individuals residing in Argentina and Brazil (Nunes et al., 2018). Currently, it is estimated that 3.7 million people have the chronic disease in Brazil (Laporta et al., 2024), with a prevalence of 1.0 to 2.4% of the population (Brasil, 2024).

According to worldwide data, the amount spent on medical care per individual can reach an annual expenditure of US$ 4,660, while the expenses can amount to U$27,684 over a lifetime (Lee et al., 2013; Quintino et al., 2020; Ferreira et al., 2022; Perissato et al., 2022). The disease can affect the cardiovascular, digestive, and nervous systems (Balouz et al., 2017), and affects vulnerable populations, being associated with poverty in low and middle-income countries (Quintino et al., 2020; Perissato et al., 2022). Among its clinical manifestations, the cardiac and digestive forms affect older adults, often leading to early retirement, which has a significant economic and social impact (Quintino et al., 2020; Perissato et al., 2022).

A CD diagnosis comes with several limitations, and the method of choice depends on the clinical phase (Candia-Puma et al., 2022; Suescún-Carrero et al., 2022). The acute phase is often asymptomatic, presenting high rates of parasitemia. Diagnosis is based on visualization of trypomastigote forms through blood smear staining, which is considered the gold standard diagnosis for acute CD (Daltro et al., 2019; PAHO, 2019). However, regardless the high parasitemia present in acute phase, the microscope observation for disease diagnosis may present variations in sensitivity, once it mainly depends on the professional’s expertise (Schijman et al., 2022). In spite of that, it is still recommended due to its accessibility and low cost (Norman and López-Vélez, 2019; PAHO, 2019). In addition to parasitological methods, the Guidelines for the Diagnosis and Treatment of Chagas Disease also recommend serological follow-up to monitor the acute phase (PAHO, 2019).

The onset of the chronic phase can last for several years or even the host’s entire life (Moser et al., 2023). The low levels of parasitemia in this phase and the high number of anti- *T. cruzi-*specific antibodies require the use of methods based on the antigen–antibody detection assays (Santos et al., 2017), such as indirect hemagglutination assay (IHA), indirect immunofluorescence assay (IHA), point-of-care (POC), and ELISA (PAHO, 2019; Celedon et al., 2021; Freitas et al., 2022). Although serological methods are recommended for diagnosing chronic CD, their performances can vary, depending on the anti- *T. cruzi* antigen preparation (Rodrigues-dos-Santos et al., 2018). Indeed, guidelines for diagnosing chronic CD require disease confirmation by at least two different serological methods (PAHO, 2019; Candia-Puma et al., 2022).

Due to the non-standardization of a reference test and the high cost to public health, efforts have been made to optimize the development of new diagnostic tests (Bern et al., 2019). New molecules have been developed with this in mind, and, among them, recombinant antigens, such as recombinant protein (RP) and recombinant multiepitope protein (RMP), have been recognized as promising for the diagnosis of several different diseases. These molecules increase the specificity and sensitivity of assays (Dipti et al., 2006; Ebrahimi et al., 2020; Lemes et al., 2022), improving CD diagnosis (Leony et al., 2019; Celedon et al., 2021; dos Santos et al., 2022). Such recombinant antigens can be produced through genetic engineering techniques in hosted cells, which allows the production of pure, highly specific proteins (Santos et al., 2017; Dopico et al., 2019; Freitas et al., 2022). Due to the promising application of recombinant antigens in CD diagnosis, the aim of this review is to discuss studies that used recombinant antigens for CD diagnosis, employing ELISA POC tests.

## 2 Advantages of using recombinant antigens in serological diagnosis

The RP market was evaluated in US$ 49.70 million in 2021, with a projected 16.9% growth rate by 2028 (Vantage Market Research, 2022; De Brabander et al., 2023). These antigens have assorted applications, including food production, beverages, renewable energy fuels, clothing, cosmetics, biopolymers, cleaning materials, waste management, and medicines (Puetz and Wurm, 2019). Additionally, the application of RPs has conquered a space within the diagnostic line, representing a significant market for new diagnostic targets for neglected diseases (Camussone et al., 2009; Casulli, 2021). RPs are proteins of native origin, without modifications in their original state (Langlais and Korn, 2006). They are produced through genetic engineering, where techniques allow their expression in heterologous host systems, such as bacteria and yeasts, obtaining them in ideal amounts with a high degree of purity (Camussone et al., 2009; Rosano and Ceccarelli, 2014; Pouresmaeil and Azizi-Dargahlou, 2023). They have been applied in the diagnosis of several diseases, such as Covid-19 (Ramos et al., 2023; Vilca-Alosilla et al., 2023), toxoplasmosis (Kotresha and Noordin, 2010), hemorrhagic fever and Ebola (Saijo et al., 2006), and leishmaniasis (Lage et al., 2023), with promising results.

RMP is a single molecule that does not exist in nature and is the product of the junction of epitopes (Dipti et al., 2006; Galdino et al., 2016). Epitope selection, one of the main points in the process of constructing a new RMP, can be performed through several methods, such as bioinformatic analyses and phage display (Hajissa et al., 2015; Mucci et al., 2017). Moreover, designing this new molecule also involves selecting the number of epitopes that will be used, selecting linking spacers, and evaluating its physical–chemical parameters (Galdino et al., 2016). RMPs contain a high density of epitopes, which improves specificity and sensitivity. Like RPs, RMPs can be obtained through expression platforms, such as yeast, bacteria, insects, and animal cells (Roberts et al., 2013; Rosano and Ceccarelli, 2014; Pollet et al., 2021; Cabal and Wu, 2022). The use of RMPs has been applied to the diagnosis of various diseases, such as CD, canine visceral leishmaniasis, toxoplasmosis, hepatitis C, dengue, and tuberculosis, with satisfactory results (Cervantes-Landín et al., 2014; Del-Rei et al., 2019; Freitas et al., 2022; Souza et al., 2022; Dias et al., 2023; Machado et al., 2023), improving sensitivity, specificity, and diagnostic accuracy (Dipti et al., 2006; Ebrahimi et al., 2020).

The use of diagnostic technologies with recombinant antigens is a promising strategy for CD diagnosis, given that these antigens improve sensitivity and specificity of a serological diagnosis. Moreover, this technology presents low-cost production and improved reproducibility, storage, and stability (Gomes et al., 2001; García-Bermejo et al., 2022). The use of these antigens has been gaining ground in CD diagnosis, showing promising results, as discussed above.

## 3 Method

For this narrative review, the search for scientific articles was carried out using the PubMed database, including all papers published up to April/2024. The descriptors used are described in Table 1. The selected articles were screened using inclusion and exclusion criteria, reviewed by two different readers. Bibliographical reviews, case studies, epidemiological reviews, molecular and serological diagnoses of other diseases, editorials, duplicate articles, and articles related to other subjects were excluded. Only those articles employing ELISA or point of care assays using recombinant proteins or multiepitope recombinant proteins for human CD diagnosis were included, regardless of whether there was a comparison with commercial tests or whether there was more than one test.

**Table 1:** Descriptors used in the PubMed search.

(chagas disease[Title/Abstract]) AND (diagnosis[Title/Abstract]) AND (recombinant [Title/Abstract])
(chagas disease[Title/Abstract]) AND (serodiagnosis[Title/Abstract])
(chagas disease[Title/Abstract]) AND (serodiagnosis[Title/Abstract]) AND (recombinant [Title/Abstract])
(chagas disease[Title/Abstract]) AND (immunodiagnosis[Title/Abstract])
(chagas disease[Title/Abstract]) AND (immunodiagnosis[Title/Abstract]) AND (recombinant [Title/Abstract])
(*Trypanosoma cruzi*[Title/Abstract]) AND (diagnosis[Title/Abstract]) AND (recombinant [Title/Abstract])
(*Trypanosoma cruzi*[Title/Abstract]) AND (serodiagnosis[Title/Abstract])
(*Trypanosoma cruzi*[Title/Abstract]) AND (serodiagnosis[Title/Abstract]) AND (recombinant [Title/Abstract])
(*Trypanosoma cruzi*[Title/Abstract]) AND (immunodiagnosis[Title/Abstract])
(*Trypanosoma cruzi*[Title/Abstract]) AND (immunodiagnosis[Title/Abstract]) AND (recombinant [Title/Abstract])

## 4 Recombinant protein-based antigens applied in CD diagnosis

### 4.1 Recombinant protein-based ELISA

The first studies for the development of ELISA techniques, developed by Swiss scientists Engvall and Perlmann, occurred around 1941 (Engvall and Perlmann, 1971). Currently, the ELISA assay is widely used as a laboratory diagnostic tool, being one of the foremost analytical tools for the development of researches in the biotechnological and biomedical areas, used to quantify specific antigens or antibodies of a given sample (Gan and Patel, 2013). The ELISA assay offers several benefits, such as improved sensitivity, specificity, and low cost, in addition to being a well-established assay (de Matos Franco et al., 2021).

Almeida et al. (1990) worked with two recombinant antigens, named FRA and CRA, which were expressed using *Escherichia coli*. A total of 221 *T. cruzi*-positive serum samples were employed to evaluate protein’s reactivity, in addition to 242 *T. cruzi*-negative serum samples. Moreover, serum samples from individuals affected with other diseases, such as leishmaniasis and malaria, were employed to evaluate cross-reactions. FRA and CRA were mixed in an ELISA assay, and a 100% of sensitivity and specificity was observed.

Gruber and Zingales (1993) examined the diagnostic efficacy of an RP named B13, which was expressed using *E. coli* DH5α cells. The serological panel included 85 *T. cruzi*-positive serum samples and 124 *T. cruzi*-negative serum samples. Results demonstrated that B13-based ELISA agreed with other serological tests, as it was able to detect all positive serum samples.

Pastini et al. (1994) developed the Dia Kit Bio-Chagas assay (Gador S.A.), which consists of a mixture of recombinant antigens. These RPs, named antigens 1 and 2, SAPA, Ag13 and Ag30 antigens, and were obtained through heterologous expression in *E. coli* HB1O1 cells. Initially, a serological panel of 52 and 122 serum samples from acute and chronic CD carriers, respectively, and 58 *T. cruzi*-negative serum samples, was used to assess the reactivity of each recombinant antigen separately. Results showed that all positive serum samples recognized at least one of the recombinant proteins, with no cross-reactivity. The kit’s performance was evaluated using 300 and 350 *T. cruzi*-positive and *T. cruzi*-negative serum samples, respectively, where it was observed a 99.6% sensitivity and 99.1% specificity.

Godsel et al. (1995) developed an RP based on a flagellar calcium-binding protein. This new recombinant protein, named FCaBP, was obtained using *E. coli* cells. To evaluate its reactivity, 18 *T. cruzi*-positive serum samples and six *T. cruzi*-negative serum samples were used. Moreover, serum samples from individuals with leishmaniasis were used to assess possible cross-reactions. The FCaBP-based ELISA results showed 100% sensitivity and specificity values.

Subsequently, Umezawa et al. (1996) worked with the B13 RP, which was also obtained using *E. coli* DH5α cells. The B13’s diagnostic performance was analyzed using 40 *T. cruzi*-positive serum samples and 20 *T. cruzi*-negative serum samples. Results showed that IgM and IgG reactivity was 55 and 65%, respectively, when using serum samples from acute CD carriers. However, when using serum samples from chronic CD carriers, B13 reactivity was 9% for IgM and 100% for IgG. B13 was less recognized by serum samples from acute and chronic CD carriers as compared to the parasite’s lipopeptido-phosphoglycan and epimastigote alkaline extract.

Umezawa et al. (1999) developed six RPs, named H49, A13, JL7, B13, JL8, and 1F8. After obtaining these antigens from *E. coli* cells, their diagnostic performance was analyzed using 541 serum samples, of which 304 were *T. cruzi*-positive serum samples and 237 were *T. cruzi*-negative serum samples, including serum samples from healthy individuals and individuals with other diseases. Sensitivity values were calculated as 97.7, 97.4, 87.1, 93.4, 93.8, and 99.0% for H49, JL7, A13, B13, JL8, and 1F8, respectively. Despite their elevated sensitivity, RPs showed reduced sensitivity compared to epimastigote-based ELISA. Specificity values were determined as 97.5, 96.6, 99.6, 99.2, 96.2, and 99.6% for H49, JL7, A13, B13, JL8, and 1F8, respectively. In contrast with their sensitivity values, the specificity values of the RPs were higher compared to the epimastigote-based ELISA.

Thomas et al. (2001) worked with an RP, named KMP11, which was expressed using *E. coli* cells. Its diagnostic efficacy was evaluated through a serological panel consisting of 20 *T. cruzi*-positive serum samples and 10 *T. cruzi*-negative serum samples. Additionally, serum samples from individuals with tuberculosis, leishmaniasis, and malaria were used for cross-reaction testing. Although KMP11 was recognized by *T. cruzi*-positive serum samples, it was also recognized by positive leishmaniasis serum samples. Sensitivity and specificity values were not provided.

Meira et al. (2002) then developed an RP, called rCRP, which was expressed in heterologous *E. coli* system cells. A serological panel of 184 samples was used to evaluate its reactivity, 65 of which were *T. cruzi*-positive serum samples and 100 *T. cruzi*-negative serum samples. In addition, serum samples from individuals with leishmaniasis were used to check for cross-reactivity. In the end, the ELISA assay showed 100% sensitivity and specificity.

Telles et al. (2003) described the use of recombinant ubiquitin antigens for CD diagnosis. After obtaining recombinant ubiquitin using *E. coli* cells, a serological panel of 104 *T. cruzi*-positive serum samples and 50 *T. cruzi*-negative serum samples were used to evaluate its diagnostic performance. Moreover, cross-reactions were checked using serum samples from individuals positive for leishmaniasis, malaria, and toxoplasmosis. Results showed 89.4% sensitivity and 93.8% specificity. They showed that ubiquitin has improved specificity compared to whole *T. cruzi* epimastigote extract. However, the sensitivity value was lower compared to the whole *T. cruzi* epimastigote extract.

Pereira-Chioccola et al. (2003) developed an RP, named TS, which was obtained using the *E. coli* system cells. Its diagnostic performance was evaluated using a serological panel containing 151 *T. cruzi*-positive serum samples and 40 *T. cruzi*-negative serum samples. Serum samples from individuals with visceral leishmaniasis and other diseases were also used to access cross-reactions. The ELISA test with the TS recombinant protein showed 98% sensitivity and specificity between 94 and 100%.

Umezawa et al. (2003) conducted a study using three RPs, named B13, 1F8, and H49, using a serological panel comprising 617 *T. cruzi*-positive serum samples and 147 *T. cruzi*-negative serum samples to evaluate their diagnostic potential. To assess possible cross-reactions, 133 serum samples from individuals infected with other diseases, such as leishmania and toxoplasmosis, were used. B13-based ELISA showed sensitivity and specificity values of 95 and 99.2%, respectively. Regarding 1F8 diagnostic performance, sensitivity, and specificity values were determined as 98.5 and 99.6%, respectively. Concerning CD diagnosis using H49, 96.6% sensitivity and 97.8% specificity were observed. Moreover, a combination of the three recombinant proteins was evaluated with Mix-based ELISA showing sensitivity and specificity values of 99.7 and 98.6%, respectively.

Umezawa et al. (2004) conducted a study using three RPs, named MAP, JL8, and TcPo. A serological panel of 180 *T. cruzi*-positive serum samples was used to evaluate recombinant recognition by positive serum samples, comprising serum samples from acute and chronic CD carriers. In addition, 80 *T. cruzi*-negative serum samples and 62 serum samples from individuals with other diseases were also used, with ELISA showing sensitivity rates of 100% for JL8, 82% for MAP, and 73% for TcPO. However, specificity values were not provided. In addition, they were put together to form what was called JM, MT, and JT mixture recombinant antigens. For serum samples from chronic CD carriers, all mixture recombinant antigens showed 100% sensitivity. Sensitivity values for serum samples from acute CD carriers were determined as 84.2, 78.9, and 84.2% for JM, MT, and JT, respectively, and specificity, values were determined as 99.3, 96.5, and 98.6%, for JM, MT, and JT, respectively.

Marcipar et al. (2005) conducted a study using three RPs, rC29FL, rC29N, and rC29c, which were obtained using *E. coli* cells. A serological panel of 68 *T. cruzi*-positive serum samples and 33 *T. cruzi*-negative serum samples was used to evaluate the diagnostic potential of these recombinant proteins. In addition, serum samples from individuals infected with other diseases were used to assess the possibility of cross-reactions. The rC29FL-based ELISA exhibited 98.5% sensitivity and 94% specificity. In terms of the diagnostic performance of rC29c, sensitivity and specificity values were 70 and 100%, respectively. Furthermore, rC29n showed sensitivity and specificity values of 98.5 and 98%, respectively.

De Marchi et al. (2011) developed an RP, named GST-TSSA VI. After obtaining it using cells from the *E. coli* system, a serological panel of 237 *T. cruzi*-positive serum samples and 200 *T. cruzi*-negative serum samples was used to assess RP’s reactivity. In addition, 180 serum samples from individuals with unrelated diseases were also used. Results showed that GST-TSSA VI presented 86.9% sensitivity and 97.4% specificity.

Valiente-Gabioud et al. (2011) evaluated the performance of three RPs, named FRA1, FRA2, and FRA4, which were obtained in *E. coli* cells. *T. cruzi*-positive serum samples were used, with the results showing that these serum samples were capable of recognizing all recombinant proteins. Moreover, the avidity of the antibodies was analyzed using 10 positive serum samples, in which antibodies showed higher avidity for the FRA4 recombinant antigen.

Vasconcelos et al. (2011) employed CRA and FRA, previously developed, in the CD serodiagnosis. A serological panel of 96 *T. cruzi*-positive serum samples was used to evaluate the diagnostic potential of these recombinant proteins. When evaluating IgM reactivity using the CRA and FRA antigens, it was observed a 10.42% of positivity when using CRA and 11.46% when employing FRA.

Longhi et al. (2012) evaluated the diagnostic performance of an RP, named JL7. In their study, a serological panel of 228 *T. cruzi*-positive serum samples and 108 *T. cruzi*-negative serum samples was used. Furthermore, serum samples from individuals affected with other diseases were used to analyze cross-reactions. JL7-based ELISA showed a sensitivity value of 95.2%, demonstrating a similar diagnostic performance to the epimastigote-based ELISA. The specificity value was calculated as 100%, which was higher than those of the epimastigote-based ELISA.

Reis-Cunha et al. (2014) conducted a study using RPs, named rTc_11623.20 and rTc_N_10421.310, which were expressed in *E. coli* cells. To assess the protein’s reactivity, 58 *T. cruzi*-positive serum samples and 55 *T. cruzi*-negative serum samples were used. Serum samples from individuals with leishmaniasis were also used to check possible cross-reactions. An rTc_11623.20-based ELISA showed 94.83% sensitivity and 98.18% specificity. Regarding rTc_N_10421.310 results, a sensitivity of 89.66% and a specificity of 94.55% was observed. Ferreira-Silva et al. (2021) evaluated the performance of the recombinant protein rCRP, previously tested by Meira et al. (2002). In their study, 29 *T. cruzi*-positive serum samples, 30 *T. cruzi*-negative serum samples, and 179 inconclusive serum samples were used. The rCRP-ELISA demonstrated a positivity of 93.1% among *T. cruzi*-positive serum samples, showing reduced effectiveness as compared to commercial kits. However, rCRP was also recognized by 26.7% of negative serum samples.

Ruiz-Márvez et al. (2020) produced an RP named Tc964 using *E. coli* M15 cells. They analyzed Tc964’s diagnostic ability using a serological panel of 63 *T. cruzi*-positive serum samples and 6 *T. cruzi*-negative serum samples. Moreover, 23 serum samples from individuals with other diseases were used to assess cross-reactions. The study demonstrated that Tc964 was recognized by most of the *T. cruzi*-positive serum samples, without a cross-reaction with any sample tested.

### 4.2 Recombinant protein-based point-of-care

POC tests were developed as a diagnostic strategy for the rapid and accurate detection of infections, being able to identify the presence or absence of a particular antibody qualitatively (Goble and Rocafort, 2017). Although the POC test has long been considered a promising strategy for diagnosing a wide range of diseases, during the COVID-19 pandemic the urgent need to develop these tests expanded, highlighting the importance of this test (Nichols, 2021; PAHO, 2021). The early results of these rapid tests have shown impressive sensitivity and specificity and represent an alternative to laboratory tests (Ortega-Arroyo et al., 2021).

Luquetti et al. (2003) assessed the diagnostic accuracy of Chembio’s Chagas STAT-PAK test (Chembio Diagnostic Systems, Medford, NY) which comprises a combination of RPs named B13, 1F8, and H49/JL7. Its performance was evaluated using a serological panel of 393 serum samples, including 200 *T. cruzi*-positive serum samples, and 150 *T. cruzi*-negative serum samples. Samples from other diseases were also used to assess cross-reactions. Chagas Stat Pak demonstrated a sensitivity and specificity of 98.5 and 94.8%, respectively. Subsequently, the test was evaluated using 352 serum samples from four Latin American countries. Among these samples, 279 samples were classified as *T. cruzi*-positive serum samples by conventional serology. Using this serological panel, 100% sensitivity and 98.6% specificity was observed.

Ponce et al. (2005) continued studies using the Chagas STAT-PAK rapid test for CD diagnosis. The test’s diagnostic performance was evaluated using a serological panel of 5,998 serum samples, including serum samples from blood donors, individuals diagnosed with cardiopathy, and serum samples received from international diagnostic laboratories. The Chagas STAT-PAK was shown to have 99.6% sensitivity and 99.9% specificity displaying an elevated agreement with results from the commercial ELISA.

Houghton et al. (2009) used two RPs, named ITC6 and ITC8.2, in a rapid test. These proteins were heterologously expressed in *E. coli* cells. The reactivity of ITC6 and ITC8.2 was evaluated separately using different serological panels: a panel of 15 sera from Venezuela, Nicaragua, Honduras, and Argentina composed of 14 *T. cruzi*-positive serum samples and 1 *T. cruzi*-negative serum sample; a panel of 21 sera from Central and South America; a serological panel of 25 *T. cruzi*-positive serum samples; a serological panel of 118 *T. cruzi*-positive serum samples from Chile; and a serological panel of 106 serum samples from non-endemic controls and individuals affected by other diseases such as toxoplasmosis, leishmaniasis, non-parasitic diseases and rheumatoid factor. The sera used for this study were obtained from Venezuela, Nicaragua, Honduras, Argentina, and Chile. The results showed that ITC8.2 presented greater sensitivity as compared to ITC6, demonstrating a 99.2% sensitivity and 99.1% specificity. Table 2 summarizes the main points of the above-cited studies.

**Table 2:** Recombinant proteins applied in CD immunodiaganosis.

Recombinant protein name	Expression platform	Serological panel (positive/negative serum samples/cross-reactions)	Test used	Results	Author/ Country
CRA and FRA	*E. coli*	221 *T. cruzi*-positive serum samples 242 *T. cruzi*-negative serum samples 8 serum samples positive for rheumatoid factor 15 serum samples positive for schistosomiasis 12 serum samples positive for malaria 10 serum samples positive for toxoplasmosis 14 serum samples positive for syphilis 21 serum samples positive for leishmaniasis	ELISA	Sensitivity: 100% Specificity: 100%	Almeida et al. (1990) / Brazil
B13	*E. coli*	85 *T. cruzi*-positive serum samples 124 *T. cruzi*-negative serum samples	ELISA	B13 showed reactivity with all positive serum samples, demonstrating similar serological performance with other serological tests	Gruber and Zingales (1993) / Brazil
Kit – Bio Chagas (Antigens 1, 2, SAPA, Ag13, and Ag20)	*E. coli*	300 *T. cruzi*-positive serum samples 350 *T. cruzi*-negative serum samples16 serum samples positive for VL	ELISA	Sensitivity: 99.6% Specificity: 99.1%	Pastini et al. (1994) / Argentina
FCaBP	*E. coli*	18 *T. cruzi*-positive serum samples 6 *T. cruzi*-negative serum samples 3 serum samples positive for leishmaniasis	ELISA	Sensitivity: 100% Specificity: 100%	Godsel et al. (1995) / USA
B13	*E. coli*	18 acute *T. cruzi*-positive serum samples 22 chronic *T. cruzi*-positive serum samples 20 *T. cruzi*-negative serum samples	ELISA	Acute phase - IgM reactivity: 55% IgG reactivity: 65% Chronic phase - IgM reactivity: 9% IgG reactivity: 100%	Umezawa et al. (1996) / Brazil
H49 A13 JL7 B13 JL 8 1F8	*E. coli*	304 chronic *T. cruzi*-positive serum samples 237 *T. cruzi*-negative serum samples 1 serum sample of positive *T. rangeli* 5 serum samples positive for toxoplasmosis 4 serum samples positive for malaria 4 serum samples positive for paracoccidioidomycosis 5 serum samples positive for syphilis 16 serum samples from patients positive for connective tissue diseases and positive for antinuclear antibodies 7 serum samples with rheumatic fever 40 serum samples positive for VL	ELISA	H49 - Sensitivity: 97.7% Specificity: 97.5% JL7 - Sensitivity: 97.4% Specificity: 96.6% A13 - Sensitivity: 87.1% Specificity: 99.6% B13 - Sensitivity: 93.4% Specificity: 99.2% JL8 - Sensitivity: 93.8% Specificity: 96.2% 1F8 - Sensitivity: 99% Specificity: 99.6%	Umezawa et al. (1999) / Brazil
KMP11	*E. coli*	20 chronic *T. cruzi*-positive serum samples 10 *T. cruzi*-negative serum samples 10 serum samples positive for leishmaniasis 5 serum samples positive for tuberculosis 5 serum samples positive for malaria	ELISA	KMP11 was recognized by all *T. cruzi*-positive serum samples (mean reactivity 1.06) and leishmaniasis positive serum samples (mean reactivity 0.87)	Thomas et al. (2001) / Spain
rCRP	*E. coli*	65 chronic *T. cruzi*-positive serum samples 100 *T. cruzi*-negative serum samples09 serum samples positive for CL 10 serum samples positive for VL	ELISA	Sensitivity: 100% Specificity: 100%	Meira et al. (2002) / Brazil
Ubiquitin	*E. coli*	10 acute *T. cruzi*-positive serum samples 94 chronic *T. cruzi*-positive serum samples 50 *T. cruzi*-negative serum samples 45 serum samples positive for CL 10 serum samples positive for VL 15 serum samples positive for ML 22 serum samples positive for malaria 20 serum samples positive for toxoplasmosis	ELISA	Sensitivity: 89.4% Specificity: 93.8%	Telles et al. (2003) / Venezuela
TS	*E. coli*	151 chronic *T. cruzi*-positive serum samples 40 *T. cruzi*-negative serum samples10 serum samples positive for VL	ELISA	Sensitivity: 98% Specificity: 94 to 100%	Pereira-Chioccola et al. (2003) / Brazil
B13 1F8 H49	*E. coli*	617 chronic *T. cruzi*-positive serum samples 147 *T. cruzi*-negative serum samples 1 serum sample positive for *T. rangeli* 5 serum samples positive for toxoplasmosis 4 serum samples positive for malaria 4 serum samples positive for paracoccidioidomycosis 5 serum samples positive for schistosomiasis 8 serum samples positive for syphilis 16 serum samples from patients positive for connective tissue diseases and positive for antinuclear antibodies 7 serum samples with rheumatic fever 80 serum samples from patients positive for VL and CL	ELISA	B13 - Sensitivity: 95% Specificity: 99.2% 1F8 - Sensitivity: 98.5% Specificity: 99.6% H49 - Sensitivity: 96.6% Specificity: 97.8% Mix of antigens - Sensitivity: 99.7% Specificity: 98.6%	Umezawa et al. (2003) / Brazil
RP combination (B13, 1F8, H49, and JL7)	*E. coli*	200 chronic *T. cruzi*-positive serum samples from Brazil and 279 *T. cruzi*-positive serum samples from different Latin American countries 150 *T. cruzi*-negative serum samplesfrom Brazil and 73 *T. cruzi*-negative serum samplesfrom different Latin American countries 9 serum samples positive for Kala-azar disease 10 serum samples positive for ML 11 serum samples positive for hepatitis B 3 serum samples positive for HIV 10 serum samples for autoimmune diseases (systemic lupus erythematosus, and scleroderma, with or without rheumatoid factor)	Immunochromatographic assay (POC)	Using serum from the same region -Sensitivity: 98.5% Specificity: 94.8% Using serum from different regions of Latin America - Sensitivity: 100% Specificity: 98.6%	Luquetti et al. (2003) / Brazil
JL8 TcPO Mix JM Mix MT Mix MJT	*E. coli*	19 acute *T. cruzi*-positive serum samples 161 chronic *T. cruzi*-positive serum samples 80 *T. cruzi*-negative serum samples 9 positive serum samples of *T. rangeli* 5 serum samples positive for toxoplasmosis 10 serum samples positive for leishmaniasis 4 serum samples positive for malaria 5 serum samples positive for paracoccidioidomycosis 5 serum samples positive for schistosomiasis 19 serum samples positive for connective tissue diseases and positive for antinuclear antibodies 5 serum samples positive for rheumatic fever	ELISA	JL8 - Sensitivity: 100% MAP - Sensitivity: 82% TcPO - Sensitivity: 73% Sensitivity of Mix antigens JM acute phase: 84.2 JM chronic phase: 100 MT acute phase: 78.9 MT chronic phase: 100 MJT acute phase: 84.2 MJT chronic phase: 100 Specificity - JM: 99.3% MT: 96.5% MJT: 98.6%	Umezawa et al. (2004) / Brazil
rC29FL rC29c rC29N	*E. coli*	68 *T. cruzi*-positive serum samples 33 *T. cruzi*-negative serum samples 15 serum samples positive for leishmaniasis	ELISA	rC29FL - Sensitivity: 98.5% Specificity: 94% rC29c - Sensitivity: 70% Specificity: 100% rC29n - Sensitivity: 98.5% Specificity: 98%	Marcipar et al. (2005) / Brazil
ITC6 ITC8.2	*E. coli*	118 *T. cruzi*-positive serum samples 106 control and positive serum samples for other diseases (toxoplasmosis, leishmaniasis, non-parasitic diseases, and rheumatoid factor)	Lateral flow immunoassay (POC)	ITC6 - Recognized by most *T. cruzi*-positive serum samples ITC8.2 - Sensitivity: 99.2% Specificity: 99.1%	Houghton et al. (2009) / USA
GST-TSSA VI	*E. coli*	237 *T. cruzi*-positive serum samples 200 *T. cruzi*-negative serum samples 29 serum samples positive for CL 31 serum samples positive for VL 4 serum samples positive for histoplasmosis 9 serum samples positive for *Mycobacterium leprae* 4 serum samples positive for *Schistosoma ssp.* 5 serum samples positive for *Giardia lamblia* 1 serum sample positive for *Hymenolepis nana* 4 serum samples positive for *Tricuris trichiura* 2 serum samples positive for *Strongyloides stercoralis* 2 serum samples positive for *Ancylostoma* spp. 3 serum samples positive for *Ascaris lumbricoides* 1 serum sample positive for *Enterobius vermicularis* 3 serum samples positive for *Toxocara canis* 3 serum samples positive for *Cryptosporidium* spp. 53 serum samples positive for rheumatoid arthritis 26 serum samples positive for systemic lupus erythematosus	Chemiluminescent ELISA	Sensitivity: 86.9% Specificity: 97.4%	De Marchi et al. (2011) / USA
FRA1 FRA2 FRA4	*E. coli*	*T. cruzi*-positive and negative serum samples	Indirect ELISA	Results showed that the RPs were able to identify *T. cruzi*-positive and negative cases	Valiente-Gabioud et al. (2011) / Argentina
CRA FRA	*–*	96 chronic *T. cruzi*-positive serum samples	Indirect ELISA	CRA – IgM positivity: 10.42% FRA – IgM positivity: 11.46%	Vasconcelos et al. (2011) / Brazil
JL7	*E. coli*	228 chronic *T. cruzi*-positive serum samples 108 *T. cruzi*-negative serum samples5 serum samples positive for VL 4 serum samples positive for ML 19 serum samples positive for autoimmune diseases 16 serum samples positive for cardiomyopathies of *T. cruzi*-negative serum samples etiology 5 serum samples positive for other diseases (juvenile diabetes, schistosomiasis, idiopathic megaesophagus, and South American blastomycosis)	ELISA	Sensitivity: 95.2% Specificity: 100%	Longhi et al. (2012) / Argentina
rTc_11623.20 rTc_N_10421.310	*E. coli*	58 *T. cruzi*-positive serum samples 45 *T. cruzi*-negative serum samples 5 serum samples positive for CL 5 serum samples positive for VL	ELISA	rTc_11623.20 - Sensitivity: 94.83% Specificity: 98.18% rTc_N_10421.310 - Sensitivity: 89.66% Specificity: 94.55% Mixed antigens - Sensitivity: 96.55% Specificity: 98.18%	Reis-Cunha et al. (2014) / Brazil
rCRP	*E. coli*	29 *T. cruzi*-positive serum samples 30 *T. cruzi*-negative serum samples 179 serum samples from inconclusive screening	In-house ELISA	Recognized by 93.1% of *T. cruzi*-positive serum samples and 26.7% of negative serum samples	Ferreira-Silva et al. (2021) / Brazil
Tc964	*E. coli*	63 *T. cruzi*-positive serum samples 6 *T. cruzi*-negative serum samples 23 serum samples positive for CT	ELISA	Recognized by most *T. cruzi*-positive serum samples	Ruiz-Márvez et al. (2020) / Colombia

CL, Cutaneous leishmaniasis; ML, mucocutaneous leishmaniasis; VL, visceral leishmaniasis; POC, point-of-care.

## 5 Multiepitope recombinant protein-based antigens applied in CD diagnosis

### 5.1 Multiepitope recombinant protein-based ELISA

Ferreira et al. (2001) reported the development and evaluation of a recombinant fusion protein, called TcF, which contains four different peptides. After obtaining TcF through *E. coli* cells, a serological panel of 101 *T. cruzi*-positive serum samples, 150 *T. cruzi*-negative serum samples blood donors, and 39 serum samples positive for leishmaniasis was used to assess protein reactivity. The TcF-based ELISA showed 100% sensitivity and 98.94% specificity.

Later, Camussone et al. (2009) developed two RPs, named CP1 and CP2. After using *E. coli* cells to obtain these proteins, antigenicity was assessed by means of a serological panel containing 141 *T. cruzi*-positive serum samples and 164 *T. cruzi*-negative serum samples. Moreover, serum samples from individuals with leishmaniasis were used to evaluate cross-reactions. Results showed that CP1 and CP2 presented a greater antigenicity as compared to the mix of peptides that comprise each one. Furthermore, CP2 showed higher diagnostic performance, demonstrating 98.6% sensitivity and 99.4% specificity.

Hernández et al. (2010) developed an RMP, named TcBDE, which was obtained using heterologous *E. coli* XL1-Blue/pREP cells. Its diagnostic effectiveness was evaluated using a serological panel containing 165 *T. cruzi*-positive serum samples and 216 *T. cruzi*-negative serum samples. TcBDE-based ELISA showed 99.3% sensitivity and 100% specificity.

Cimino et al. (2011) developed an RMP, named rTSSA-II. The RMP’s antigenicity was evaluated using 41 *T. cruzi*-positive serum samples and *T. cruzi*-negative serum samples. *T. cruzi*-positive serum samples co-infected with leishmaniasis were also used. Positive serum samples from individuals with only leishmaniasis were used to assess cross-reactions. The results showed that rTSSA II was recognized by 92.24% of *T. cruzi*-positive serum samples. Moreover, specificity was determined as 100%. Regardless such good results, rTSSA II’s sensitivity was inferior compared to a commercial test.

Pierimarchi et al. (2013) continued the studies with the TcF antigen, using a serological panel of 55 *T. cruzi*-positive serum samples and 77 *T. cruzi*-negative serum samples to further evaluate TcF reactivity. Results showed 98% sensitivity and 100% specificity. Later, Duthie et al. (2016) developed two RMPs, named TcF43 and TcF26, expressed in *E. coli* cells. To evaluate the protein’s reactivity, 286 *T. cruzi*-positive serum samples and 96 serum samples from healthy individuals were used. Results showed that TcF43 and TcF26 proteins increased serum recognition as compared to antigens used in commercial kits. However, sensitivity and specificity values were not provided.

Santos et al. (2016) developed four new RMPs, named IBMP-8.1, IBMP-8.2, IBMP-8.3, and IBMP-8.4. After obtaining the antigens using *E. coli* system cells, the RMP’s performance was evaluated using serum samples from 20 *T. cruzi*-negative serum samples and 280 *T. cruzi*-positive serum samples. IBMP-8.1-based ELISA showed 98.9% sensitivity and 100% specificity. The sensitivity and specificity values of IBMP-8.2 were determined as 98.2 and 90%, respectively, whereas the IBMP-8.3 results showed 95.4% sensitivity and 95% specificity. Regarding IBMP8-4-based ELISA, sensitivity and specificity values were calculated as 99.6 and 100%, respectively.

Continuing the studies with the IBMP RMPs, Santos et al. (2017) conducted a phase II study to evaluate the accuracy of these antigens. In their study, the antigens’ performance was evaluated using 825 and 630 *T.cruzi*-positive and *T. cruzi*-negative serum samples, respectively. Moreover, serum samples from individuals with other diseases, such as leishmaniasis, were used. Results showed that IBMP-8.4 had the greatest sensitivity and specificity values, estimated as 99.3 and 100%, respectively. IBMP-8.1, IBMP.8–2, and IBMP-8.3 sensitivity values were determined as 97.4, 94.3, and 97.9%, respectively. In addition, specificity values were calculated as 99.4, 99.6, and 99.9% for IBMP-8.1, IBMP-8.2, and IBMP-8.3, respectively.

Next, Daltro et al. (2019) carried out a detailed analysis of the IBMP’s cross-reactivity. In this regard, 600 serum samples from American cutaneous leishmaniasis and 229 serum samples from visceral leishmaniasis were analyzed. All the samples were collected in leishmaniasis-endemic regions in the northeastern states of Brazil, including Bahia, Pernambuco, and Rio Grande do Norte. When considering all positive leishmaniasis serum, this study reported that the IBMP chimeric antigens exhibited minimal cross-reactivity, with its incidence calculated as 2.4% for IBMP-8.1, 4.7% for IBMP-8.2, 1.3% for IBMP-8.3, and 1.7% for IBMP-8.4. IBMP-based ELISA showed reduced cross-reactions as compared to the commercial immunoassays.

Continuing these studies, Dopico et al. (2019) used a serological panel containing 347 *T. cruzi*-positive serum samples and 331 *T. cruzi*-negative serum samples. Furthermore, cross-reactions were assessed using serum samples from individuals infected with *Toxoplasma gondii* and the Zika virus. The sensitivity and specificity values of IBMP-8.1 were determined to be 99.4 and 100%, respectively. Regarding the IBMP-8.4 results, 99.1% sensitivity and 99.7% specificity were observed.

A study conducted by Freitas et al. (2022) continued evaluating the diagnostic capacity of IBMP-8.1, IBMP-8.2, IBMP-8.3, and IBMP-8.4. In their study, 207 *T. cruzi*-positive serum samples and 205 *T. cruzi*-negative serum samples were used. In addition, leishmaniasis, hepatitis, HTLV-1/2, HIV-1/2, and syphilis serum samples were used to assess possible cross-reactions. Sensitivity values were determined as 74.4, 87, 88.4, and 79.2% for IBMP-8.1, IBMP-8.2, IBMP-8.3, and IBMP-8.4, respectively. IBMP-8.1, IBMP-8.2, and IBMP-8.4 showed a 100% specificity value, while IBMP-8.3 demonstrated 96.6% specificity.

dos Santos et al. (2022) evaluated the diagnostic capabilities of IBMP-8.1, IBMP-8.2, IBMP-8.3, and IBMP-8.4 in a serological screening. A total of 5,014 serum samples from blood donors were used, of which 21 and 4.993 serum samples were classified as *T. cruzi*-positive and *T. cruzi*-negative, respectively. IBMP-8.4 showed the highest sensitivity value, calculated as 100%, followed by IBMP-8.3 (95.24%), IBMP-8.2 (90.48%), and IBMP-8.1 (85.71%). IBMP-8.1 and IBMP-8.2 antigens demonstrated the highest specificity values, determined as 100%, while IBMP-8.3 and IBMP-8.4 had values of 99.98%.

Machado et al. (2023) developed an RMP, named rTC, obtained using *E. coli* cells. For serological reactivity analysis, a total of 58 *T. cruzi*-positive serum samples was used. In addition, 30 *T. cruzi*-negative serum samples were used as negative control, and serum samples from diseases that could present cross-reactions, such as visceral and cutaneous leishmaniasis, were also used. Results showed that rTC had sensitivity and specificity value of 98.28 and 96.67%, respectively.

Lastly, Santos et al. (2023) evaluated the cross-reactivity of the IBMP-8.1, IBMP-8.2, IBMP-8.3, and IBMP-8.4 antigens. For this purpose, seven *Crithidia* sp. LVH-60A- positive serum samples and three *Leishmania infantum*-positive serum samples were employed. Regarding cross-reactivity with *Crithidia* sp. LVH-60A- positive serum samples, none of the antigens demonstrated reactivity with these samples, with 20% of samples falling in the gray zone for IBMP-8.2 and IBMP-8.4 antigens, while 40% of samples fell within the gray zone for IBMP-8.3. Concerning *L. infantum*-positive serum samples, IBMP-8.1 antigen demonstrated a 33.3% cross-reactivity. In addition, 33.3% of samples fell within the gray zone when analyzing IBMP-8.4 antigen.

### 5.2 Multiepitope recombinant protein-based point-of-care

The IBMP-8.1 and IBMP-8.4, widely tested in ELISA, were also employed in lateral flow assay for CD diagnosis. The study was conducted by Silva et al. (2020), where 16 *T. cruzi* positive-serum samples, and 16 *T. cruzi* negative-serum samples, were used to evaluate the antigens’ performance in the lateral flow assay. Results showed that both antigens showed a 100% accuracy, detecting all positive serum samples, aside from not presenting false-positive results when analyzing *T. cruzi* negative-serum samples.

Medina-Rivera et al. (2022) assessed the diagnostic accuracy of a newly developed lateral flow assay, AuNS-LFA, using a chimeric recombinant protein containing four *T. cruzi* antigens, PEP-2, TcD, TcE, and SAPA, immobilized on chromatographic strips. A total of 42 serum samples, comprising 15 *T. cruzi*-positive serum samples and 27 *T. cruzi*-negative serum samples were used to evaluate the test’s performance. Results showed that the combination of four antigens had 83% sensitivity and 95% specificity demonstrating an inferior performance compared to a commercial test. Table 3 summarizes the main points of these studies.

**Table 3:** Recombinant multiepitope proteins applied in CD immunodiaganosis.

Recombinant multiepitope proteins	Expression platform	Serological panel (positive/negative serum samples/cross-reactions)	Test used	Results	Author/ Country
TcF	*E. coli*	101 *T. cruzi*-positive serum samples 150 *T. cruzi*-negative serum samples 39 serum samples positive for leishmaniasis	ELISA	Sensitivity: 100% Specificity: 98.94%	Ferreira et al. (2001) / Brazil
CP1 CP2	*E. coli*	141 *T. cruzi*-positive serum samples 164 *T. cruzi*-negative serum samples 15 serum samples positive for leishmaniasis	ELISA	CP1 showed good discrimination efficiency using *T. cruzi*-positive serum samples CP2 - Sensitivity: 98.6% Specificity: 99.4%	Camussone et al. (2009) / Brazil
TcBDE	*E. coli*	165 *T. cruzi*-positive serum samples 216 *T. cruzi*-negative serum samples 50 serum samples positive for syphilis 35 serum samples positive for leishmaniasis	ELISA	Sensitivity: 99.3% Specificity: 100%	Hernández et al. (2010) / Germany
rTSSA -II	*E. coli*	41 *T. cruzi*-positive serum samples 37 serum samples positive for both *T. cruzi* and CL 54 *T. cruzi*-negative serum samples 79 serum samples positive for CL	ELISA	92.24% positivity rate Specificity: 100%	Cimino et al. (2011) / Argentina
TcF	*E. coli*	55 *T. cruzi*-positive serum samples 77 *T. cruzi*-negative serum samples	ELISA	Sensitivity: 98% Specificity: 100%	Pierimarchi et al. (2013) / Brazil
TcF43 TcF26	*E. coli*	286 *T. cruzi*-positive serum samples 96 *T. cruzi*-negative serum samples	ELISA	TcF43 and TcF26 were strongly recognized by *T. cruzi*-positive serum samples	Duthie et al. (2016) / USA
IBMP-8.1 IBMP-8.2 IBMP-8.3 IBMP-8.4	*E. coli*	280 chronic *T. cruzi*-positive serum samples 20 *T. cruzi*-negative serum samples	In-house ELISA	IBMP-8.1 - Sensitivity: 98.9% Specificity: 100% IBMP-8.2 - Sensitivity: 98.2% Specificity 90% IBMP-8.3 - Sensitivity: 95.4% Specificity: 95% IBMP-8.4 - Sensitivity: 99.6% Specificity: 100%	Santos et al. (2016) / Brazil
IBMP-8.1 IBMP-8.2 IBMP-8.3 IBMP-8.4	*E. coli*	825 chronic *T. cruzi*-positive serum samples 630 *T. cruzi*-negative serum samples50 serum samples positive for dengue 51 serum samples positive for filariasis 163 serum samples positive for hepatitis B 98 serum samples positive for hepatitis C 140 serum samples positive for HIV 109 serum samples positive for HTLV 153 serum samples positive for leishmaniasis 92 serum samples positive for leptospirosis 21 serum samples positive for measles 15 serum samples positive for rubella 42 serum samples positive for schistosomiasis 145 serum samples positive for syphilis	In-house ELISA	IBMP-8.1 - Sensitivity: 97.4% Specificity: 99.4% IBMP-8.2 - Sensitivity: 94.3% Specificity: 99.6% IBMP-8.3 - Sensitivity: 97.9% Specificity: 99.9% IBMP-8.4 - Sensitivity: 99.3% Specificity: 100%	Santos et al. (2017) / Brazil
IBMP-8.1 IBMP-8.2 IBMP-8.3 IBMP-8.4	*E. coli*	600 chronic positive serum samples positive for CL 229 serum samples positive for VL	In-house ELISA	IBMP-8.1 - Cross-reaction: 2.4% IBMP-8.2 - Cross-reaction: 4.7% IBMP-8.3 - Cross-reaction: 1.3% IBMP-8.4 - Cross-reaction: 1.7%	Daltro et al. (2019) / Brazil
IBMP-8.1 IBMP-8.4	*E. coli*	347 chronic *T. cruzi*-positive serum samples 331 *T. cruzi*-negative serum samples98 serum samples positive for *Toxoplasma gondii* 75 serum samples positive for Zika virus	In-house ELISA	IBMP-8.1 - Sensitivity: 99.4% Specificity: 100% IBMP-8.4 - Sensitivity: 99.1% Specificity: 99.7%	Dopico et al. (2019) / Brazil
IBMP-8.1 IBMP-8.4	*E. coli*	16 *T. cruzi* positive serum samples 16 *T. cruzi* negative serum samples	Lateral flow assay (POC)	IBMP-8.1 - 100% diagnostic accuracy IBMP-8.4 - 100% diagnostic accuracy	Silva et al. (2020) / Brazil
IBMP-8.1 IBMP-8.2 IBMP-8.3 IBMP-8.4	*E. coli*	207 chronic *T. cruzi*-positive serum samples 205 *T. cruzi*-negative serum samples 10 serum samples positive for leishmaniasis 20 serum samples positive for hepatitis B 10 serum samples positive for hepatitis C 9 serum samples positive for HTLV-1/2 9 serum samples positive for HIV-1/2 10 serum samples positive for syphilis	Double-antigen sandwich ELISA	IBMP-8.1 - Sensitivity: 74.4% Specificity: 100% IBMP-8.2 - Sensitivity: 87% Specificity: 100% IBMP-8.3 - Sensitivity: 88.4% Specificity: 96.6% IBMP-8.4 - Sensitivity: 79.2% Specificity: 100%	Freitas et al. (2022) / Brazil
IBMP-8.1 IBMP-8.2 IBMP-8.3 IBMP-8.4	*E. coli*	21 chronic *T. cruzi*-positive serum samples 4,993 *T. cruzi*-negative serum samples	ELISA	IBMP-8.1 - Sensitivity: 85.71% Specificity: 100% IBMP-8.2 - Sensitivity: 90.48% Specificity: 100% IBMP-8.3 - Sensitivity: 95.24% Specificity: 99.98% IBMP-8.4 - Sensitivity: 100% Specificity: 99.98%	dos Santos et al. (2022) / Brazil
AuNS -LFA (PEP2, TcD, TcE and SAPA)	*E. coli*	15 chronic *T. cruzi*-positive serum samples 27 *T. cruzi*-negative serum samples	Lateral flow assay (POC)	Sensitivity: 83% Specificity: 95%	Medina-Rivera et al. (2022) / USA
rTC	*E. coli*	58 chronic *T. cruzi*-positive serum samples 30 *T. cruzi*-negative serum samples 30 serum samples positive for CL 30 serum samples positive VL	In-house ELISA	Sensitivity: 98.28% Specificity: 96.67%	Machado et al. (2023) / Brazil
IBMP-8.1 IBMP-8.2 IBMP-8.3 IBMP-8.4	*E. coli*	7 *Crithidia* sp. LVH-60A-positive serum samples 3 L. *infantum-*positive serum samples	Indirect ELISA	*Crithidia* sp. LVH-60A-positive serum samples - No cross-reactions were observed. 20% of samples fell within the gray zone for IBMP-8.2 and IBMP-8.4 antigens; while 40% of samples fell within the gray zone for IBMP-8.3 analyses L. *infantum-*positive serum samples - 33.3% cross-reactivity was observed for IBMP-8.1, while 33.3% of samples fell within the gray zone for IBMP-8.4 analyses.	Santos et al. (2023) / Brazil

CL, Cutaneous leishmaniasis; VL, visceral leishmaniasis; ML, leishmaniasis mucocutaneous; POC, Point of care.

## 6 Discussion

In recent years, CD has evolved in social, economic, and environmental terms (Lidani et al., 2019). Until the 20th century, CD was mainly associated with areas considered extremely poor. However, the disease currently affects American, European, and Asian countries (Echeverría et al., 2020). It is known that CD imposes a very high financial cost on the health system (Andrade et al., 2023; de Sousa et al., 2024), in addition to having a profound effect on the infected individual’s life. For infected individuals to have successful treatment, they must receive comprehensive care, starting with an early *T. cruzi* infection diagnosis (Pérez-Molina and Molina, 2018). The acute phase is characterized by the occurrence of symptoms that are considered nonspecific, and, at this stage, serological diagnostic methods are usually not indicated to detect the disease, making it necessary to develop a serological test with elevated sensitivity since there is a low production of antibodies at this stage (Ortega-Arroyo et al., 2021). In spite of that these limitations, serological tests for detecting the disease in the acute phase are already commercially available, such as the IFI Chagas Disease Bio-Manguinhos (Rio de Janeiro, RJ, Brazil), demonstrating optimistic progress in the serological diagnosis of this disease. Moreover, although serological tests are considered an initial technique for diagnosis during the chronic phase, they have some limitations, such as varying levels of sensitivity and specificity. In this sense, facing the heterogeneity of test accuracy (Marchiol et al., 2023; López et al., 2024; Rivero et al., 2024), the need to confirm the disease through at least two serological tests generates more costs to the public health system. In that regard, there is an urgent need to develop new diagnostic tests to detect the disease in the chronic and acute phases.

Recombinant antigens, such as RPs and RMPs, have been widely used and offer such benefits as purity, high specificity, sensitivity, scalability, and cost-effectiveness. These advantages make them beneficial tools in the development of diagnostic assays (Ricci et al., 2023). To improve the CD serological diagnosis, several researchers have expanded efforts to develop new tests based on these antigens. Indeed, there has recently been an increased use of recombinant antigens in CD diagnostic studies. The data summarized above indicates that most of the studies using RPs had sensitivity and specificity above 90%. Similarly, studies using RMPs for CD diagnosis also showed a high diagnostic capacity. However, despite the excellent performance of both recombinant antigens, it is not possible to infer which antigen type would be better for diagnosing CD, mainly due to the different serological panels used, different geographical locations of the studies, and different protocols applied. Nonetheless, it is important to highlight that, over time, some of these RPs and RMPs were tested in different studies, demonstrating their diagnostic potential. Antigens such as B13, JL7, TSSA, FRA, TcF, IBMP-8.1, IBMP-8.2, IBMP-8.3 and IBMP-8.4 showed promising results in the majority of the studies in which they were tested. In fact, given the promising results, some of these antigens are part of commercial tests already developed, such as IgG-ELISA^®^ (NovaTec Immunodiagnostica GmbH; Dietzenbach, Germany), Chagas ELISA IgG + IgM^®^ (Vircell^®^, Granada, Spain), Chagas Detect™ Plus (CDP) Rapid Test (InBios International Inc., Seattle, United States) and Chagas Stat-Pak (Chembio Diagnostic Systems Inc., New York, USA). Despite good results with RP and RMPs as described in previous studies, there are some important points that need to be improved. During the process of developing new diagnostic kits, it is important to consider the *T. cruzi’*s genetic variability, which is considered one of the most important factors that could interfere with the safety and quality of diagnostic results (Ribeiro et al., 2024). It is known that different *T. cruzi* strains can be found in different geographic areas. These different environments can influence the parasite’s protein expression, which can, in turn, affect the test’s accuracy based on recombinant antigens. To minimize this problem, the use of conserved and immunodominant antigens (Dipti et al., 2006) is a strategy that aims improving the diagnostic performance of the same test in different regions. In addition to the parasite’s genetic variability, the host’s genetic variability is also an important point to consider as a once-immune response can vary according to factors such as diet, nutritional status, genetic influence, secondary diseases, and disease history (Andrade et al., 2014; Ribeiro et al., 2024). To assess the real sensitivity of the test in response to different factors, multicenter studies need to be developed (Iturra et al., 2023), primarily associated with statistical programs to calculate the sample number and obtain more reliable results. Indeed, most of the studies mentioned above used a small sample size, requiring new studies with broader testing. Furthermore, the development of new specific diagnostic kits for both the acute and chronic phases is seen as essential. The vast majority of above-referenced studies used sera from the chronic phase in their tests, leaving a gap in testing acute-phase sera. It could be hypothesized that the low-level testing of acute phase sera is linked to the lack of access to sera from individuals at this stage, considering that endemic areas are usually developing countries that have limited access to information and health and medical services. In addition, the symptomatology in this phase is not specific, which may contribute to delays in seeking medical care at the onset of the disease, making an early and specific diagnosis difficult. Some measures must be taken into consideration when developing new CD serological tests. For example, it is essential that one use low-cost reagents, designed for better accessibility and more widespread testing. Furthermore, the development and implementation of rapid tests are essential to providing a rapid and reliable diagnosis, even in remote areas where access is difficult and nearby specialized laboratories are scarce. Moreover, the use of different types of samples, such as saliva and urine, could simplify testing as they are less invasive. The need to improve ELISA techniques should also be highlighted, aimed at providing more accurate and reproducible diagnoses, thus reducing the number of tests. In addition, the development of new biomarkers that could allow monitoring the disease progression would greatly assist in clinical decision-making. Finally, in addition to the points mentioned above, it is also necessary to strengthen bonds between public and private research with the public health system. It is known that better control or even eradication of several diseases, such as CD, will probably only be possible with collaborations, whether between researchers or between research and the public health system. In this sense, aiming to establish a more practical and accurate diagnosis for the disease, more incentives from government agencies are needed to encourage as well as facilitate research, strengthening bonds and creating a bridge between innovation and the implementation of these innovations. In summary, this review affirms that the use of recombinant antigens has shown encouraging results when it comes to the need for a better CD diagnosis. Therefore, there is need to conduct ongoing studies in search of new antigens to develop faster, more precise, and more effective diagnostic methods.

## Author contributions

CR: Data curation, Investigation, Methodology, Validation, Writing – original draft, Writing – review & editing, Visualization. AR: Data curation, Methodology, Validation, Writing – original draft, Writing – review & editing. IG: Data curation, Methodology, Visualization, Writing – original draft, Writing – review & editing. KS: Methodology, Validation, Visualization, Writing – review & editing. LL: Methodology, Validation, Visualization, Writing – review & editing. IB: Methodology, Validation, Visualization, Writing – review & editing. CC: Validation, Visualization, Writing – review & editing, Methodology. MF: Methodology, Validation, Visualization, Writing – review & editing. SP: Methodology, Visualization, Writing – review & editing. SX: Methodology, Validation, Visualization, Writing – review & editing. JM: Conceptualization, Methodology, Supervision, Validation, Visualization, Writing – review & editing. MP: Methodology, Supervision, Validation, Visualization, Writing – review & editing. AC: Methodology, Validation, Visualization, Writing – review & editing. EC: Methodology, Validation, Visualization, Writing – review & editing. RG: Methodology, Supervision, Validation, Visualization, Writing – review & editing. MC-F: Methodology, Supervision, Validation, Visualization, Writing – review & editing. WD: Conceptualization, Formal analysis, Methodology, Supervision, Validation, Visualization, Writing – review & editing. AnG: Conceptualization, Formal analysis, Investigation, Methodology, Supervision, Validation, Visualization, Writing – original draft, Writing – review & editing. AlG: Conceptualization, Investigation, Project administration, Supervision, Validation, Writing – original draft, Writing – review & editing, Data curation, Formal analysis, Funding acquisition, Methodology, Resources, Visualization.
